# Is postoperative transanastomotic feeding beneficial in neonates with congenital duodenal obstruction?

**DOI:** 10.1007/s00383-021-05053-3

**Published:** 2021-12-15

**Authors:** Martin Treider, Anders Hauge Engebretsen, Hans Skari, Kristin Bjørnland

**Affiliations:** 1 Oslo University Hospital Department of Gastro and Pediatric Surgery, Oslo University hospital HF, Nydalen, Postboks 4950, 0424 Oslo, Norway; 2 University of Oslo, Problemveien 7, 0315 Oslo, Norway

**Keywords:** Transanastomotic feeding tube, Duodenal obstruction, Neonatal surgery, ERAS, Annular pancreas, Enteral feeding

## Abstract

**Purpose:**

We aimed to evaluate possible positive and negative effects of postoperative use of transanastomotic feeding tube (TAFT) in neonates operated for congenital duodenal obstruction (CDO).

**Methods:**

This is a retrospective study reviewing medical records of neonates operated for CDO during 2003–2020 and comparing postoperative feeding outcomes and complications in patients with and without TAFT. Approval from the hospital’s data protection officer was obtained.

**Results:**

One hundred patients, 59% girls, were included, and 37% received TAFT. Mean birth weight and gestational age were 2628 (675.1) grams and 36.6 (2.4) weeks, respectively. Furthermore, 45% had no other malformations, and 36% had Down syndrome. Patient demographics were similar for TAFT and not-TAFT patients, except that not-TAFT neonates weighed median 335 g less (*p* = 0.013). The TAFT group got parenteral nutrition 2 days shorter (*p* < 0.001) and started enteral feeds 1.5 days earlier (*p* < 0.001) than the not-TAFT group. Fewer neonates with TAFT got a central venous catheter [65 vs 89%, (*p* = 0.008)]. In the TAFT group, 67% were breast fed at discharge compared to 49% in the not-TAFT group (*p* = 0.096).

**Conclusion:**

Neonates with TAFT had earlier first enteral feed, fewer days with parenteral nutrition and fewer placements of central venous catheters.

## Introduction

Congenital duodenal obstruction (CDO) is most commonly caused by duodenal atresia, duodenal web, or obstructive annular pancreas, and affects about 0.5–1.5 in 10 000 newborns [1–3]. Standard treatment is surgery within the first few days after birth, creating a duodenoduodenostomy [1, 4]. One of the main challenges after CDO surgery is enteral feeding due to gastric and duodenal retention caused by dysmotility in the dilated stomach and proximal duodenum. Almost all patients need some form of nutritional support the first days after surgery, either as parenteral nutrition (PN) or enteral nutrition through a nasogastric or transanastomotic feeding tube (TAFT). A nasogastric tube is usually inserted in addition to the TAFT to aspirate contents from the stomach and duodenum that do not pass the anastomosis in the immediate postoperative period. The use of TAFT in neonates with CDO varies, and rates from 2 to 100% are reported [1, 5–10]. It seems that surgeons’ preference is the main determinant for placement of a TAFT or not. The latest research indicates many benefits and few disadvantages with TAFT [5, 7, 8, 11]. Despite this, a recent large study showed that only 42% of neonates received TAFT [1].

From a physiological point of view, enteral feeding is preferred over PN as it maintains gastrointestinal function and avoids PN-associated complications. Furthermore, enteral feeding is cost-effective compared to PN. It has been reported that TAFT can reduce health expenses by increasing enteral nutrition and reducing expensive PN [5]. TAFT may also reduce time to full enteral feeds, less need for a central venous catheter (CVC), and shorter postoperative length of stay [11]. However, not all studies have been able to demonstrate these benefits of TAFT [5, 7]. Furthermore, complications related to TAFT have been reported, and these include tube dislodgement, clogging, and bowel perforation. Dislodgement has been reported in about 14% of cases, but bowel perforation is rare [5, 7, 12].

As evidence for the advantages and disadvantages of TAFT is limited [13], the present study aims to gain more knowledge about the effects of TAFT in neonates undergoing surgical treatment for CDO. The main aim was to investigate whether patients with TAFT had fewer postoperative days with PN. Secondary aims were to assess whether TAFT reduced the need for CVC, if there were any TAFT-related complications, and if TAFT interfered with breastfeeding.

## Methods

This is a retrospective study reviewing medical records of neonates undergoing surgery for CDO at Oslo University hospital between 2003 and 2020. CDO was defined as a complete or incomplete obstruction of the bowel between the pylorus and the ligament of Treitz. The obstruction was categorized as either duodenal web, duodenal atresia, annular pancreas, or other (tumor or malrotation). Patients were identified by electronic search for the ICD-10 diagnosis “Q41.0 Congenital duodenal atresia or stenosis” and “Q45.1 Annular pancreas” in the medical records database, and manually in the surgical logbooks. Data were collected from electronic medical records and registered in EpiData Manager (version 4.6.0.2).

Patients’ demographics, comorbidity, gestational age, age at surgery, and time of diagnosis (pre- or post-natally) were registered. Additional malformations were grouped as congenital heart disease, gastrointestinal malformations, and other malformations. To be categorized as congenital heart disease, the cardiac disease required need for surgical, medical, or radiological intervention during hospitalization and/or need for follow-up after discharge. Perioperative data such as type of operation and TAFT placement and postoperative parameters including length of stay, time to full preanastomotic feeding (oral and/or nasogastric tube feeding), postoperative days with PN, time to first breastfeed, and 30-day mortality were recorded. Postoperative growth was estimated by calculating the difference between weight at the day of operation and weight at discharge and then dividing that on the number of postoperative days in our institution. Postoperative complications were graded according to the Clavien–Dindo (CD) classification system, and major complications were defined as CD 3 or more [14].

Oslo University Hospital is a regional surgical center for neonatal surgery severing a population of approximately 3,000,000, and patients with complex heart conditions are referred from the whole country (serving approximately 5,350,000 patients). The TAFT used in our institution was a Charriere 5 or 6 silicon feeding tube, inserted in one nostril, and placed distal to the ligament of Treitz. TAFT was placed in CDO patients according to the surgeon’s preference. In addition, a nasogastric tube was usually placed through the other nostril or mouth. Babies were given breast milk, either from their mother or from the hospital’s breast milk bank unless there were particular reasons for not giving breast milk. In all patients both with and without TAFT, the amount of preanastomotic feeding (nasogastric tube, breast feeding or bottle feeding) was adjusted according to aspirate volumes and the neonate’s tolerance. There was no defined protocol for postoperative feeding, preanastomotic feeding was reduced if the child vomited, showed discomfort or if aspirate volumes excided 30–60 ml. The attending surgeon prescribed enteral and parenteral nutrition daily according to the clinical status of the neonate. Moreover, the TAFT was removed when full preanastomotic feed was achieved.

For statistical analyses, SPSS statistics 23, (IBM corp. Armonk, NY) was used. Patients were divided into two groups, those who received TAFT (TAFT group), and those who did not (not-TAFT group). Furthermore, patient characteristics and data on preoperative treatment were analyzed to address potential differences between the groups. Then, postoperative outcomes were analyzed to assess the possible implications of TAFT. Categorical variables are presented as the number of patients and percentages. Normally distributed data were presented as mean (standard deviation), and non-normally distributed data as median (min–max). Statistical significance was analyzed with Mann–Whitney *U* test, Student *t* test, and chi-square test for non-parametric numbers, parametric numbers, and categorical variables, respectively.

The study was approved by the Data Protection Officer (20/13386).

## Results

One hundred and one patients fulfilled the inclusion criteria. One patient was excluded because the medical record was not retrievable, leading to a total of 100 patients. Fifty-nine (59%) patients were girls. Mean birth weight and gestational age at birth were 2628 (675.1) grams and 36.6 (2.4) weeks, respectively. Furthermore, 45 (45%) patients had no other malformation, and 36 (36%) patients were diagnosed with Down Syndrome. The median age at operation was two (0–24) days and all surgeries were performed as laparotomies. Thirty-one (31%) had duodenal web, 34 (34%) duodenal atresia, 30 (30%) annular pancreas, one (1%) tumor and one (1%) malrotation. Median postoperative stay in our institution was 14 (5–111) days, and 63% were discharged to their local hospital. During the study period, 37 (37%) patients got TAFT. The rate of neonates getting TAFT has been relatively stable during the study period with 32.2% during 2003–2008, 28.9% during 2009–2014, and 52% during 2015–2020 (*p* = 0.130). The 30-day mortality was 3%, all occurred in the not-TAFT group, and all deaths were caused by serious cardiac disease.

The TAFT and the not-TAFT groups were similar in terms of patient characteristics and operative treatment except for lower birth weight in the not-TAFT group (Table 1). The lowest birth weight was 1545 g in the TAFT group and 1400 g in the not-TAFT group. The TAFT group got PN for two fewer postoperative days than the not-TAFT group (*p* < 0.001) and started enteral feeding 1.5 postoperative days earlier (*p* < 0.001) (Table 2). Moreover, 35% of the patients with TAFT did not get a CVC, compared to 11.1% in the not-TAFT group (*p* < 0.008). Neonates receiving a CVC did not have a higher rate of Down Syndrome (*p* = 0.610), gastrointestinal malformations (*p* = 0.790) or heart disease (*p* = 0.794) than those not receiving a CVC. Ten percent of all CVCs were peripheral inserted central venous catheters. No notable differences between the groups were found in terms of time to full preanastomotic feeding, CVC-related infections, breastfeeding at discharge, or growth during the hospital stay (Table 2).

**Table 1:** Patient characteristics of 100 neonates operated for congenital duodenal obstruction grouped as transanastomotic feeding tube (TAFT) or not-TAFT

	TAFT (*n* = 37)	Not-TAFT (*n* = 63)	*p* value
Age at operation, days (median, min–max)	2 (0–19)	2 (0–24)	0.608
Gender, girls	23 (62.5%)	36 (57.5%)	0.677
Gestational age at birth, weeks (mean, SD)^a^	37.1 (2.4)	36.4 (2.7)	0.208
Birth weight, grams (mean, SD)	2839 (642.4)	2504 (667.9)	0.013
Prematurity^a^	15 (42.9%)	27 (42.9%)	1.00
Additional malformations			
None	20 (54.1%)	25 (39.7%)	0.212
Heart	11 (29.7%)	24 (38.1%)	0.515
Gastrointestinal^b^	7 (18.9%	21 (33.3%)	0.167
Other	2 (5.4%)	7 (11.1%)	0.478
Down syndrome	15 (40.5%)	21 (33.5%)	0.521
Type of obstruction			
Duodenal web	15 (40.5%)	16 (25.2%)	0.123
Duodenal atresia	10 (27.8%)	24 (39.3%)	0.278
Annular pancreas	11 (30.6%)	19 (31.1%)	1.000
Other (tumor and malrotation)	0 (0%)	2 (3.3%)	0.528
Type of operation^a^			
Duodenoduodenostomy	33 (89.2%)	51 (81.0%)	0.399
Duodenojejunostomy	4 (10.8%)	7 (11.1%)	1.000
Duodenotomy	0 (0%)	3 (4.8%)	0.154
Other (Ladd’s procedure and gastroduodenostomy)	0 (0%)	2 (2.8%)	0.529
Patients undergoing concomitant procedures^c^	11 (29.7%)	27 (42.9%)	0.730

^a^Value was missing in one or more patients for this variable

^b^Including Meckel’s diverticulum, malrotation, anorectal malformations and esophageal atresia

^c^Including Ladd’s procedure, esophagoesophagostomy, resection of Meckel’s diverticulum, appendectomy, anal dilatation, gastrostomy, peritoneal dialysis catheter and sigmoid stoma

**Table 2:** Postoperative outcomes in 100 neonates operated for congenital duodenal obstruction grouped as transanastomotic feeding tube (TAFT) or not-TAFT

	TAFT (*n* = 37)	Not-TAFT (*n* = 63)	*p* value
No CVC	13 (35.0%)	7 (11.1%)	0.008
CVC infections (positive blood culture)	2/24 (8.3%)	4/56 (7.1%)	1.000
Postoperative days until first enteral feed^a^	1 (0–10)	2.5 (1–10)	< 0.001
Postoperative days until first breastfeed^a^	10 (5–16)	8 (5–20)	0.120
Breastfeeding started before discharge	25 (67.4%)	31 (49.2%)	0.096
Postoperative days until full preanastomotic feed^a^	10 (5–21)	9 (6–71)	0.598
Postoperative days with parenteral nutrition in our institution^a^	8 (0–17)	10 (5–71)	< 0.001
Parenteral nutrition at discharge^a^	3 (8.1%)	17 (27.4%)	0.036
Median postoperative growth, grams per day (median, max–min)^a^	19.5 (-14–93)	22.52 (-14–204)	0.304
Length of stay in our institution, days^a^	13 (7–30)	14 (5–111)	0.912
Discharged to local hospital	20 (51.1%)	43 (68.2%)	0.101

All values are given as median postoperative days (min–max)

^a^Value was missing in one or more patients for this variable

Postoperative complications occurred in 40 (40%) neonates, 23 (62.2%) in the TAFT group and 17 (27%) in the not-TAFT group (*p* < 0.001) (Table 3). The majority of complications were CD grade one TAFT-related complications such as clogging or dislodgement. Unplanned removal of the TAFT (seven by the patient, six due to dislodgement, two due to clogging) occurred after a median of six (1–14) days (Table 3). There was no difference in the rate of CD grade 2–5 complications between the groups (Table 3). One patient in the TAFT group experienced a leak in the duodenoduodenostomy. The TAFT in this patient soon dislodged into duodenum oral to the anastomosis, and therefore the TAFT was removed after 1 day. The leak was found 3 days after TAFT removal when a small defect in the anastomosis was detected, and this was closed with one suture. In addition, this patient had a cardiac arrest immediately before the reoperation, and the patient was successfully resuscitated. All deaths within 30 days were related to major heart comorbidity.

**Table 3:** 30-day complications graded according to Clavien–Dindo classification listed as number of complications in 100 neonates operated for duodenal obstruction grouped as transanastomotic feeding tube (TAFT) or not-TAFT. Ten patients had 2 or more complications

	TAFT (*n* = 37)	Not-TAFT (*n* = 63)	*p* value
Number of patients with complications	23 (62.2%)	17(27.0%)	< 0.001
Grade 1 complications	19 (51.4%)	3 (4.7%)	< 0.001
Atelectasis	2	1	
Would infection	2	2	
TAFT related^b^	15	0	
Grade 2	11 (29.7%)	16 (25.3%)	0.473
CVC infection	2	4	
Wound infection	6	6	
Other^c^	3	6	
Grade 3b	2 (5.4%)	2 (3.2%)	0.625
Anastomotic leak^a^	1	1	
Pleural fluid	1	0	
Hematemesis	0	1	
Grade 4b (cardiac arrest)	1 (2.7%)	0 (0%)	0.370
Grade 5 (death)	0 (0%)	3 (4.8%)	0.294

^a^One Anastomotic leak was in relation to a resected Meckel diverticulum and not the duodenoduodenostomy

^b^Clogging, dislodgement and unintentional removal

^c^Blood transfusions, sepsis or pneumonia

## Discussion

The main findings in this study are that the neonates getting a TAFT during CDO surgery received PN significantly shorter in our institution and started enteral feeds significantly earlier than neonates not getting TAFT. That TAFT may reduce the need for PN has been shown previously [5, 7, 11]. Only one old study has shown poorer feeding outcomes in neonates receiving TAFT [15]. Even though the TAFT group got less PN, we could not demonstrate that time to full preanastomotic feeding was not shorter for patients with TAFT, contrary to what was shown in some other studies [8, 11]. There may be several reasons for the lack of differences in time to full preanastomotic feeding in the two groups. Postoperatively, the focus is to reduce PN and to increase enteral feeding. Moreover, it is possible that giving enteral nutrition through the TAFT leads to less aggressive preanastomotic feeding. Another reason may be that dysmotility in the dilated stomach and preanastomotic duodenum prevents a faster increase in preanastomotic feeding regardless of TAFT use.

Fewer neonates in the TAFT group got a CVC during their hospital stay. Reducing the need for CVC is an important advantage as CVC-related complications are frequent and may be serious [16]. Previous research is conflicting on whether TAFT reduced the rate of CVC placements [5, 7]. Despite a reduction in CVC placements in the TAFT group this study was not able to demonstrate a reduction in CVC-related infections in the TAFT group due to a low number of patients with CVC infections.

As reported by others [5, 7], we placed a nasogastric tube in addition to the TAFT. One may worry that TAFT interferes with breastfeeding due to the presence of two tubes. However, this study could not demonstrate any difference between the groups in terms of breastfeeding at discharge or days to first breastfeed. This study is the first to investigate breastfeeding in neonates with TAFT.

The total complication rate in this study was higher than what is reported in most other studies in patients with CDO [1, 3, 17]. We think the most likely explanation is that we applied the CD classification system where all complications, including CD grade one complications, are registered. Moreover, minor CD grade 1–2 complications are rarely registered in retrospective studies [1, 5]. Most of the minor complications were related to the TAFT, and we observed a slightly higher rate of unplanned removal of the TAFT compared to previous studies [5, 7]. This may be explained by positioning the TAFT not distal enough from the ligament of Treitz, insufficient flushing of the tube to prevent clogging, and/or poor attachment of the tube. Due to the results of this study, we have recently changed routines and now flush the TAFT with water twice daily. One patient in the TAFT group with anastomotic leakage had dislodgment and removal of the TAFT on the first postoperative day. Although the time from dislodgment to recognition of the leak was 3 days, we cannot exclude that the TAFT contributed to the anastomotic leak. A similar sequence of events has been described by Hall et al. [7]. Thus, anastomotic leak needs to be considered as a rare, but possible complication to use of TAFT.

Enhanced recovery after surgery (ERAS) has lately gained interest in neonatal gastrointestinal surgery. The first ERAS consensus in neonatal gastrointestinal surgery was published in 2020 [18], and promising results have been shown [19, 20]. One of the cornerstones of ERAS is early enteral nutrition, less parenteral nutrition, and less invasive measures such as CVC. Our results show that using TAFT in neonates with CDO is in accordance with these principles.

The strength of this study is that it was conducted in a large population and almost all eligible patients were included. Thus, the study population is representative of patients undergoing CDO surgery in a referral center for neonatal surgery. All data are retrieved from electronic medical records containing detailed information from nurses, doctors, and other health care workers. Data were collected by one researcher that had not been involved in the treatment of the patients, and therefore, was not biased. In addition, it is a strength that despite the retrospective design the groups are similar in all demographic and preoperative data analyzed except from birthweight. There are also some limitations to this study. Due to the retrospective design, there is a possibility of systematic differences between the groups affecting the results, and minor complications may have been missed. In our institution, nurses write detailed reports three times a day, reducing the risk of missing complications. As mentioned earlier, birth weight was different between groups. However, the difference in birth weight was not accompanied by a difference in gestational age or prematurity. Therefore, we do not think that the difference in birth weight is important for the interpretation of results. Some patients were discharged to their local hospital, and we do not have all clinical information after discharge. Furthermore, significantly fewer patients were discharged with PN in the TAFT group than in the not-TAFT group. Since 15 patients had the TAFT removed earlier than planned the full effect of TAFT could not be assessed in these patients. Moreover, it is likely that the use of PN in the TAFT group would have been even less if the TAFTs had been used for enteral nutrition until full preanastomotic feeding as intended. We did not register feeding and aspirate volumes. Thus, we cannot exclude a possible difference in preanastomotic feeding between the groups.

To conclude, this study demonstrates earlier first enteral feed, fewer days with PN, and fewer CVC’s placed in neonates getting a TAFT compared to those not receiving a TAFT. Moreover, rates of major complications were similar. The difference between the groups was minor and further studies are needed to decide whether TAFT should be recommended in patients undergoing surgery for CDO.

## References

[1] Bethell GS, Long AM, Knight M, Hall NJ (2020). Congenital duodenal obstruction in the UK: a population-based study. Arch Dis Child Fetal Neonatal Ed.

[2] Best KE, Tennant PW, Addor MC, Bianchi F, Boyd P, Calzolari E (2012). Epidemiology of small intestinal atresia in Europe: a register-based study. Arch Dis Child Fetal Neonatal Ed.

[3] Gfroerer S, Theilen TM, Fiegel HC, Esmaeili A, Rolle U (2019). Comparison of outcomes between complete and incomplete congenital duodenal obstruction. World J Gastroenterol.

[4] Miscia ME, Lauriti G, Lelli Chiesa P, Zani A (2019). Duodenal atresia and associated intestinal atresia: a cohort study and review of the literature. Pediatr Surg Int.

[5] Harwood R, Horwood F, Tafilaj V, Craigie RJ (2019). Transanastomotic tubes reduce the cost of nutritional support in neonates with congenital duodenal obstruction. Pediatr Surg Int.

[6] Bishay M, Lakshminarayanan B, Arnaud A, Garriboli M, Cross KM, Curry JI (2013). The role of parenteral nutrition following surgery for duodenal atresia or stenosis. Pediatr Surg Int.

[7] Hall NJ, Drewett M, Wheeler RA, Griffiths DM, Kitteringham LJ, Burge DM (2011). Trans-anastomotic tubes reduce the need for central venous access and parenteral nutrition in infants with congenital duodenal obstruction. Pediatr Surg Int.

[8] Arnbjörnsson E, Larsson M, Finkel Y, Karpe B (2002). Transanastomotic feeding tube after an operation for duodenal atresia. Eur J Pediatr Surg.

[9] Kumar P, Kumar C, Pandey PR, Sarin YK (2016). Congenital duodenal obstruction in neonates: over 13 years' experience from a single centre. J Neonatal Surg.

[10] Smith MD, Landman MP (2019). Feeding outcomes in neonates with trisomy 21 and duodenal atresia. J Surg Res.

[11] Jiang W, Lv X, Xu X, Geng Q, Zhang J, Tang W (2015). Early enteral nutrition for upper digestive tract malformation in neonate. Asia Pac J Clin Nutr.

[12] Upadhyay V, Sakalkale R, Parashar K, Mitra SK, Buick RG, Gornall P (1996). Duodenal atresia: a comparison of three modes of treatment. Eur J Pediatr Surg.

[13] Biradar N, Gera P, Rao S (2021). Trans-anastomotic tube feeding in the management of congenital duodenal obstruction: a systematic review and meta-analysis. Pediatr Surg Int.

[14] Dindo D, Demartines N, Clavien PA (2004). Classification of surgical complications: a new proposal with evaluation in a cohort of 6336 patients and results of a survey. Ann Surg.

[15] Mooney D, Lewis JE, Connors RH, Weber TR (1987). Newborn duodenal atresia: an improving outlook. Am J Surg.

[16] Ullman AJ, Marsh N, Mihala G, Cooke M, Rickard CM (2015). Complications of central venous access devices: a systematic review. Pediatrics.

[17] Dalla Vecchia LK, Grosfeld JL, West KW, Rescorla FJ, Scherer LR, Engum SA (1998). Intestinal atresia and stenosis: a 25-year experience with 277 cases. Arch Surg.

[18] Brindle ME, McDiarmid C, Short K, Miller K, MacRobie A, Lam JYK (2020). consensus guidelines for perioperative care in neonatal intestinal surgery: enhanced recovery after surgery (ERAS(®)) society recommendations. World J Surg.

[19] Peng Y, Xiao D, Xiao S, Yang L, Shi H, He Q (2021). Early enteral feeding versus traditional feeding in neonatal congenital gastrointestinal malformation undergoing intestinal anastomosis: a randomized multicenter controlled trial of an enhanced recovery after surgery (ERAS) component. J Pediatr Surg.

[20] Xu L, Gong S, Yuan LK, Chen JY, Yang WY, Zhu XC (2020). Enhanced recovery after surgery for the treatment of congenital duodenal obstruction. J Pediatr Surg.

